# Mesoporous Amorphous Bulk Material Electrodes for Ultrahigh Volumetric Capacity Electrochemical Capacitors

**DOI:** 10.1002/advs.202300727

**Published:** 2023-05-03

**Authors:** Lun Li, Changjin Guo, Shuangbao Wang, Wanbiao Hu

**Affiliations:** ^1^ Key Laboratory of LCR Materials and Devices of Yunnan Province National Center for International Research on Photoelectric and Energy Materials School of Materials and Energy Yunnan University Kunming 650091 P. R. China; ^2^ Electron Microscopy Center Yunnan University Kunming 650091 P. R. China

**Keywords:** electrochemical capacitor, ion/electron transport, mesoporous amorphous bulk;ultrahigh volumetric capacity

## Abstract

Retaining satisfactory electrochemical performances under high‐mass electrode‐active‐matter loadings is important for energy storage. However, the performance decreases with increasing mass loadings due to a reduction in the ion/electron transport. In this study, a novel mesoporous amorphous bulk (MAB) material strategy is proposed. Co‐based hydroxide KCo_1.3_(OH)_3.6_ is directly electro‐deposited on the Ni foam for cathode. Comprehensive structural characterizations confirm the mesoporous, amorphous, and bulk features for KCo_1.3_(OH)_3.6_. The fabricated whole MAB‐KCo_1.3_(OH)_3.6_@Ni electrode exhibits an ultrahigh full volumetric capacity (123.7 mAh cm^−3^) with high KCo_1.3_(OH)_3.6_ mass loading (11.7 mg cm^−2^) and excellent cycling stability. Along with the MAB‐KCo_1.3_(OH)_3.6_, the mesoporous amorphous features enable fast ion diffusion and provide sufficient electroactive sites for redox reactions. In addition, the bulk nature not only facilitates the electron mobility but also guarantees structural and chemical stability. Therefore, the proposed MAB strategy and explored KCo_1.3_(OH)_3.6_ material demonstrate considerable prospects for designing electrode materials and practical applications.

## Graphical abstract

This study establishes an innovative mesoporous amorphous bulk strategy realized in KCo_1.3_(OH)_3.6_ to demonstrate the ultrahigh volumetric capacity of electrochemical capacitors with the aim of increasing ion/electron transport, decreasing the dead‐volume, ensuring cycling stability, and retaining the sound electrochemical performance of electrodes at high mass loadings.

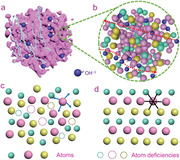

## 1 Introduction

Electrochemical capacitors have emerged as an important type of energy storage and conversion in terms of electrochemical redox reactions, and have received increasing attention due to their high‐power density, excellent cycling stability, and safety.^[^ ^1^, ^2^, ^3^, ^4^ ^]^ However, they are limited by their low energy density and are currently mainly used as backup sources in energy storage systems.^[^ ^5^, ^6^, ^7^ ^]^ Recently, a high energy density has been achieved in Ni–Co layered double hydroxide nanosheets (188 Wh kg^−1^ at 1499 W kg^−1^)^[^ ^8^ ^]^ and active ion‐tunnel‐oriented BaCoF_4_ (147 Wh kg^−1^ at 1025 W kg^−1^)^[^ ^9^ ^]^ electrodes. However, these electrodes suffer from low mass loadings of the electroactive matter, such as electrode materials (generally ≈3 mg cm^−2^), which severely restricts the gross energy capacity. Conversely, increasing the mass loading by, for example, improving the electrode film thickness through coating more electroactive matter on the current collector inevitably causes the capacitive performance to deteriorate. Thus, the mass loading of electrochemical capacitors severely restricts their practical applications.

Generally, a typical mass loading of 8–10 mg cm^−2^ is required to provide commercial devices with a feasible amount of energy.^[^ ^10^ ^]^ However, under such a high mass loading, nearly all the electrode material systems currently explored are subject to a considerably undermined electrochemical redox reaction due to inefficient electron transport and insufficient ion diffusion.^[^ ^10^, ^11^, ^12^, ^13^, ^14^ ^]^ Constructing nanostructures with large specific areas, such as hierarchical structures,^[^ ^10^ ^]^ nanoarrays,^[^ ^15^, ^16^, ^17^ ^]^ and hollow structures,^[^ ^18^ ^]^ has been used as an effective way to achieve a high electron mobility and shorten the pathways for ion diffusion. However, this would often result in a low volumetric energy density (VED) due to the dead‐volume inside the nanostructures.^[^ ^19^, ^20^, ^21^, ^22^ ^]^ Thus, the active matter loading cannot be too large (generally less than 5 mg cm^−2^) to prevent the performances, particularly that of the energy density, from being too lowered. Moreover, nanostructures are generally unstable in surface configurations during electrochemical redox reactions because the active matter could participate in the chemical reactions. This would likely cause the determined electrochemical mechanisms to be inaccurate because the surface restructuring, namely the surface chemical reactions, might play a more crucial role in the apparent performances than the real redox reactions. Consequently, there would not be a structure‐stable material or substantial methodology that would enable sufficient electron/ion transport for reaching high performances at high electroactive matter‐loadings.

**Figure 1:**
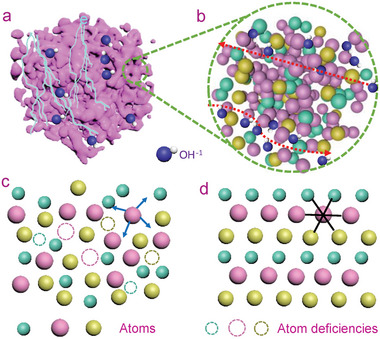
Proposed mesoporous amorphous bulk (MAB) strategy. a) The mesoporous bulk nature maintains the structural continuity for unimpededly delivering electrons (light blue) and considerably enhances the effective electroactive sites. b) Amorphous configuration facilitates the ion diffusion in the inner bulk (dashed arrows show the diffusion pathway for the OH^−^ species). c) Atomic disordering of an amorphous matter. The disordered configuration enables atom fluctuations that promote the electrochemical kinetics and cycling stability. The atomic deficiencies provide possibilities for local atom position exchanges and enhance the electrochemical redox reactions. d) Atomic ordering of crystalline matter. The atoms in crystalline matters are limited to the strictly atomic and bonding integrity and periodicity.

To meet the necessary requirements for the development of an improved electrochemical capacitor, such as simultaneously having a high electrochemical activity and high structural stability under a high mass loading, a new form of electrode material produced using a mesoporous amorphous bulk (MAB) material strategy is proposed in this study (**Figure** **1**). First, the bulk feature will have structural and chemical stability, and it will be able to maintain the structural (chemical bonds) continuity for unimpededly delivering electrons during redox reactions (Figure 1a).^[^ ^23^, ^24^ ^]^ Second, the mesoporous structure could considerably enhance the active sites (cations) for fulfilling effective redox reactions by providing sufficient inner surfaces to electrochemically react with OH^−^ species in an electrolyte (Figure 1a).^[^ ^25^, ^26^, ^27^ ^]^ Finally, the amorphous configuration, which is opposite to the ordered lattices, is subject to considerable structural/chemical/defect fluctuations, including structural disordering, bonding distortion, and atomic deficiency, that could facilitate the OH^−^ ion diffusion in the inner bulk (Figure 1b)^[^ ^28^, ^29^, ^30^, ^31^ ^]^ and, more importantly, the fluctuations themselves could serve as electroactive sites that greatly promote the electrochemical kinetics and cycling stability (Figure 1c). Moreover, amorphous configurations provide a great possibility for local atomic position exchanges to enhance redox reactions without reducing bulk and mesoporous stability. By contrast, crystalline matters, which are limited to the strictly atomic and bonding integrity and periodicity, do not possess the aforementioned features (Figure 1d).

Along with the MAB material strategy, a Co‐based hydroxide, namely KCo_1.3_(OH)_3.6_, is in situ fabricated directly on the Ni foam for cathode electrode (see Experimental Section in Supporting Information). Co was used since it has been extensively explored and recognized and possesses an intrinsically high redox ability. K^+^ is introduced to enhance the intrinsic conductivity of the electron/ion mobile ability by widening the van der Waals gap. The hydrophobic hydroxyl group considerably facilitates the transportation of OH^−^ species in the electrolyte toward the mesopores and induces their further diffusion into the amorphous bulk for redox reactions to occur. MAB‐KCo_1.3_(OH)_3.6_ displays a high areal specific capacity (Areal SC) (1.74 mAh cm^−2^), high volumetric capacity (Vol. SC) (123.7 mAh cm^−3^), and excellent cycling stability.

## 2 Results

### 2.1 Bulk, Mesoporous, and Amorphous Features of KCo_1.3_(OH)_3.6_

**Figure 2:**
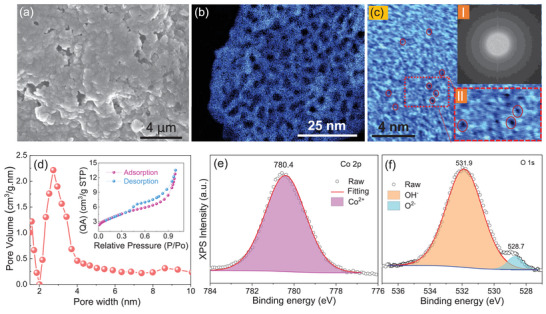
Structural and valence state characterizations of MAB‐KCo_1.3_(OH)_3.6_. a) SEM, b) TEM, and c) HR‐TEM images. Insets I and II in (c) show the selected area electron diffraction pattern and a locally enlarged HRTEM, respectively. Deficiencies are highlighted with red circles. d) Pore‐size distribution and N_2_ adsorption–desorption isotherms measured by a BET method. e,f) XPS spectra of Co 2p and O 1s for MAB‐KCo_1.3_(OH)_3.6_.

The in situ grown KCo_1.3_(OH)_3.6_ on the Ni slice (KCo_1.3_(OH)_3.6_@Ni) was comprehensively characterized. The composition was determined through an ICP‐AES coupled element analysis (Table S1, Supporting Information) and a thermogravimetry method. These techniques were also used to verify the structural stability of the necessary oxyhydrogen group in KCo_1.3_(OH)_3.6_ (Figure S1, Supporting Information). The particle size is relatively large as it generally exceeds 10 µm (**Figure** **2**a and Figure S2, Supporting Information), which indicates a bulk material feature. The mesoporous structure was confirmed by TEM‐HAADF (Figure 2b). It clearly presents the uniformly distributed mesopores with a pore size of ≈2–4 nm (Figure S3, Supporting Information) and corresponds to the Barrett–Joyner–Halenda desorption pore size distribution, which reveals an average pore size of 3 nm (Figure 2d). A specific area of 98 m^2^ g^−1^ was obtained through an N_2_ adsorption–desorption BET method (Figure 2d‐inset). HRTEM‐BF verifies the amorphous natures characterized by the disordered atomic arrangement and vague selected area electron diffraction ring (Figures 2c‐inset I and Figure S4, Supporting Information). Furthermore, there exists a significant atomic deficiency that might be due to cation defects and/or vacancies (Figures 2c‐inset II and Figure S5, Supporting Information).^[^ ^32^ ^]^ In addition, from the powder XRD pattern (Figure S6, Supporting Information), an average structure of the dominant amorphous nature could be determined and the X‐ray absorption fine structure spectra present the atomic configurations in terms of the Co K‐edge Fourier transform k^3^ *x* (k) of MAB‐KCo_1.3_(OH)_3.6_ and crystalline Co(OH)_2_ for comparison. The results exhibit the short‐range ordering and long‐range disordering of the atomic configurations of MAB‐KCo_1.3_(OH)_3.6_ (Figure S7, Supporting Information), belonging to an amorphous material. In conclusion, the in situ grown KCo_1.3_(OH)_3.6_ on the Ni slice demonstrates a simultaneously bulk, mesoporous, and amorphous feature.

XPS was performed to examine the valence states of the synthesized KCo_1.3_(OH)_3.6_. The spectra are cautiously fitted using a series of 20Lorentzian‐80Gaussian profiles. Co demonstrates a highly symmetrical 2p^3/2^ profile at 780.4 eV, which is indicative of a sole oxidation of Co^2+^ without any Co^3+^/Co^4+^. The O spectrum contains only a small amount of O^2‐^ species at 528.7 eV, while the majority of O corresponds to the binding energy of OH^−^ at 531.9 eV and is consistent with the hydrated structure.^[^ ^33^ ^]^ In addition, the MAB‐KCo_1.3_(OH)_3.6_ presents a full absorption in the visible‐light region from the UV‐vis spectrum (Figure S8, Supporting Information), which indicates that it has excellent electronic conduction.

### 2.2 Superior Electrochemical Property

**Figure 3:**
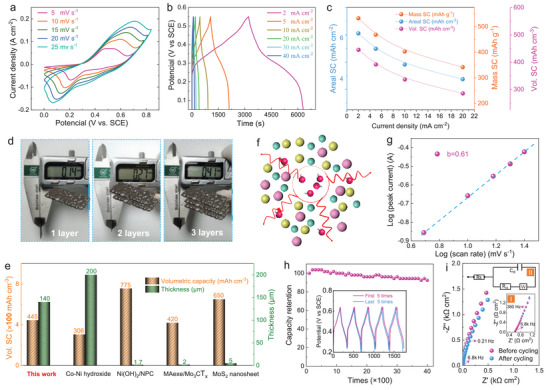
Electrochemical properties of the MAB‐KCo_1.3_(OH)_3.6_@Ni electrode. a) Cyclic voltammetry curves at different scan rates. b) Galvanostatic charge/discharge (GCD) curves at different current densities. c) Volumetric capacity (Vol. SC, mAh cm^−3^), mass specific capacity (Mass SC, mAh g^−1^), and areal specific capacity (Areal SC, mAh cm^−2^) properties as a function of the current density. d) A thickness demonstration of whole MAB‐KCo_1.3_(OH)_3.6_@Ni electrodes with 1, 2, and 3 layers. e) Diagram comparing the Vol. SC and thickness of the MAB‐KCo_1.3_(OH)_3.6_@Ni electrode with previously reported high Vol. SC electrodes. f) Schematic diagram of the OH^−^ diffusion in the inner bulk KCo_1.3_(OH)_3.6_. g) Log(*i*) versus log(v) plot of the cathodic current density response. h) Cycling stability performances at 10 mA cm^−2^. The inset shows the first and last 5 cycles of the GCD curve. i) Electrochemical impedance spectra before and after cycling 4000 times. The high‐frequency part is shown in inset I and the equivalent circuit is shown in inset II. Some frequency points (arrows) are also indicated.

To evaluate the performance of MAB‐KCo_1.3_(OH)_3.6_ as an electrode material, electrochemical tests were performed using a three‐electrode system in 6 m KOH aqueous electrolyte, in which Pt and Hg/HgO were used as the counter and reference electrodes, respectively. Notably, a relatively high mass loading of 11.7 mg cm^−2^ was used. **Figure** **3**a shows CV curves at various scan rates in the range 5–25 mV s^−1^ in a potential window of 0–0.8 V, all of which exhibit a similar shape even at a high scan rate of 25 mV s^−1^ (Figure 3a). In addition, all the galvanostatic charge–discharge curves have the same voltage plateau of 0.2–0.35 V (Figure 3b), indicating an excellent electrochemical reversibility and high‐rate performance. The apparent redox peaks present a typical battery‐type characteristic.^[^ ^34^, ^35^, ^36^ ^]^ These broad redox peaks and uniformly distributed area in the CV curves might be associated with the atomic disordering‐induced discrepant coordination environment and efficient improvement of the ion/electron transport ability that induced electrochemical redox reactions to occur at discrepant potentials and valence changes in Co^2+^→Co^3+^→Co^4+^.^[^ ^34^, ^35^, ^37^ ^]^

The Areal SCs were evaluated based on galvanostatic charge/discharge (GCD) curves (Figure 3b). An ultrahigh Areal SC and high mass specific capacity (Mass SC) of 1.74 mAh cm^−2^ and 148 mAh g^−1^, respectively, were achieved at a current density of 2 A cm^−2^. When the current density was increased to 20 A cm^−2^, both the Areal and Mass SCs decreased to 1.1 mAh cm^−2^ and 94.8 mAh g^−1^, respectively (Figure 3c). Notably, the current Areal SC was superior to nearly all the existing oxide and/or hydroxide systems with relatively high mass loadings that have been previously reported, such as MnO_2_ hierarchical nanostructures (0.75 mAh cm^−2^ at 3 mA cm^−2^),^[^ ^10^ ^]^ NiCo_2_O_4_ nanosheet‐assembled flowers on Ni foam (1.3 mAh cm^−2^ at 8 mA cm^−2^),^[^ ^38^ ^]^ and *β*‐Ni(OH)_2_/nickel‐cobalt layered double hydroxides coupled with fluorine‐modified graphene (0.55 mAh cm^−2^ at 1 mA cm^−2^).^[^ ^39^ ^]^

From the aforementioned results, it was concluded that high mass loadings would significantly reduce the specific capacity due to the inadequate delivery of ions and electrons.^[^ ^34^, ^35^, ^36^ ^]^ However, even with a high mass loading (11.7 mg cm^−2^), MAB‐KCo_1.3_(OH)_3.6_ still possesses a high Mass SC (148 mAh g^−1^ at 2 mA cm^−2^), which could be comparative with high‐performance but low‐mass‐loading Co(OH)_2_ systems, such as NiCo_2_O_4_@*α*‐Co(OH)_2_ core–shell nanowires (161.1 mAh g^−1^ at 1 A g^−1^; 1.33 ± 0.38 mg cm^−2^),^[^ ^40^ ^]^ layered *α*‐Co(OH)_2_ nanocones (146.5 mAh g^−1^ at 1 A g^−1^; 1–2 mg cm^−2^),^[^ ^41^ ^]^ and Co(OH)_2_ thin films (150.4 mAh g^−1^ at 0.83 A g^−1^; 3 mg cm^−2^).^[^ ^42^ ^]^

The Vol. SCs were further evaluated. To demonstrate a full‐volume electrode performance, the volume of a current collector foam Ni slice with a KCo_1.3_(OH)_3.6_ loading of 11.7 mg cm^−2^ (KCo_1.3_(OH)_3.6_@Ni) was considered. A whole KCo_1.3_(OH)_3.6_@Ni electrode has a more consistent thickness of 0.13–0.14 mm (Figure 3d). The packaged capacitor device with a whole KCo_1.3_(OH)_3.6_@Ni electrode (KCo_1.3_(OH)_3.6_ loading of 835.7 mg cm^−3^, total mass loading of 11.7 mg cm^−2^, and thickness of 0.13–0.14 mm) achieved an ultrahigh volume capacity of 123.7 mAh cm^−3^ (Figure 3c,e). Notably, similar or superior performances have been previously reported, such as 416.6 mAh cm^−3^ in MXene Ti_3_C_2_T*_x_* electrodes^[^ ^43^ ^]^ and 283.9 mAh cm^−3^ in the 3D structures of LSG‐MnO_2_ electrodes.^[^ ^44^ ^]^ However, the Vol. SCs of these electrodes do not take into consideration the current collector, namely the foam Ni, which occupies more than 80% of the total volume, and only the active material layer.

Furthermore, when compared with those considering both the active material and current collector, the present MAB‐KCo_1.3_(OH)_3.6_@Ni still demonstrates many significant advantages. The Vol. SC of the advanced material systems recently reported is shown in Figure 3e as a function of the electrode thickness. Fisher et al. reported an excellent capacity of 85 mAh cm^−3^ in a hierarchical Ni–Co hydroxide. However, the current collector (graphene) is expensive and contributes a part of the stored energy.^[^ ^45^ ^]^ In contrast, no energy storage is contributed by the current collector (Ni) in the MAB‐KCo_1.3_(OH)_3.6_@Ni system. Jang et al. fabricated an Ni(OH)_2_/nanoporous Au (current collector) electrochemical capacitor that exhibited a Vol. SC of 339.6 mAh cm^−3^ and considerably decreased to 215.3 mAh cm^−3^ when the thickness was increased from 0.47 to 1.7 µm. This is because the achieved high capacity must be subject to a low number of active‐material films, in which the nanoporous Au current collector improves the electron delivery but plays a poor role in ion transport.^[^ ^46^ ^]^ Similarly high capacities of 116.7 mAh cm^−3^ in a 2 µm‐thick MXene Mo_3_C_2_T*_x_* film^[^ ^47^ ^]^ and 180.6 mAh cm^−3^ in metallic 1T‐phase MoS_2_ nanosheets^[^ ^48^ ^]^ were also reported (Figure 3e). Notably, these nanostructures, such as hierarchical structures,^[^ ^10^ ^]^ nanoarrays,^[^ ^15^, ^16^, ^17^ ^]^ hollow structures,^[^ ^18^ ^]^ or nanostructure‐assembling systems,^[^ ^38^, ^49^ ^]^ suffer from low VEDs because of the dead‐volume inside the active materials. In conclusion, the MAB‐KCo_1.3_(OH)_3.6_@Ni electrode exhibits excellent electrochemical properties that are comparable or superior to those of state‐of‐the‐art material/electrode systems that have been previously reported.

Ultrahigh Vol. SCs are rarely achieved by only relying on surface reactions. Thus, a fully ionic infiltration through mesoporous channels (Figures 1b and 2b,d) and enhanced electrons delivery in structurally continuous bulk KCo_1.3_(OH)_3.6_ (Figures 1a and 2a)^[^ ^23^, ^24^ ^]^ could significantly contribute to the achieved high performances for reaching effective redox reactions by fully utilizing the active materials in KCo_1.3_(OH)_3.6_. To identify the charge storage kinetics, the diffusion behaviors were studied in terms of the log(*i*) versus log(*ν*), which is illustrated using Equation (1):^[^ ^1^, ^50^, ^51^ ^]^

(1) $$i=av^{b}$$

A *b* value of 0.5 indicates that the current is controlled by semi‐infinite diffusion, whereas a *b* value of 1 indicates a capacitive behavior.^[^ ^1^, ^50^, ^51^ ^]^ The log fitting of Equation (1) yields a *b* value of 0.61 (Figure 3g), suggesting a diffusion‐dominated nature of kinetics. However, the charge process is still slightly affected by the capacitive process because the thermodynamic balance is determined by the charge acceptance and charge potential change that would arise from the capacitance. This means that the MAB strategy could efficiently improve the ion/electron transport ability and/or decrease the timescale for the ion/electron transport. This could occur in an ultrathin LiCoO_2_ (thickness of 6 nm), where the pseudocapacitive behavior governs the diffusion within a very limited timescale.^[^ ^34^ ^]^ As a result, the main energy storage for MAB‐KCo_1.3_(OH)_3.6_ is internal diffusion storage‐dominated. In other words, the process should belong to a battery‐type behavior. Furthermore, the superior diffusion capability of amorphous KCo_1.3_(OH)_3.6_ can promote the rapid diffusion of OH^−^ (Figure 3f) for the electrochemical redox reaction.^[^ ^28^, ^29^, ^30^, ^31^ ^]^ Therefore, the cycling stability was further investigated with a device test at a current density of 5 mA cm^−2^ (Figure 3h). A high capacity retention of 92% was maintained for 4000 cycles, which is superior to most Co(OH)_2_‐based electrochemical capacitors, such as asymmetric electrochemical capacitors with bud‐like Co(OH)_2_ that only possess a capacity retention of 72.4% after 1000 cycles at 18 mA cm^−2^.^[^ ^52^ ^]^ The Mass SC of NO_3_^−^‐*α*‐Co(OH)_2_ and benzoate‐*α*‐Co(OH)_2_ after 2000 cycles at 2 A g^−1^ rapidly decreased to 81% and 72%, respectively.^[^ ^41^ ^]^ The capacitive retention of the Co(OH)_2_ microplate is only 74.6% after 6000 cycles at 1.2 A g^−1^.^[^ ^53^ ^]^ The excellent cycling stability is attributed to the amorphous and bulk feature. Fluctuations in the amorphous materials could serve as electroactive sites that considerably promote cycling stability,^[^ ^54^ ^]^ in which the bulk feature could ensure structural and chemical stability. The nearly unchanged bulk morphology after 4000 cycles confirmed that the bulk feature guarantees the stability of MAB‐KCo_1.3_(OH)_3.6_ (Figure S2c,d, Supporting Information)_._

Electrochemical impedance spectroscopy (EIS) was performed to gain insights into the electron/ion transport behavior before and after cycling at the frequency range of 0.01 Hz to 100 kHz with an open circuit potential of 5 mV (Figure 3i). The obtained Nyquist plots before and after 4000 cycles were similar. Both exhibited a low‐frequency slope line. However, an interception on Z′ and a small semicircle (Figure 3i‐inset I) was observed at a relatively high frequency before and after cycling, indicating excellent cycling stability. A general equivalent circuit (Figure 3i‐inset II) was used to fit the EIS curves, where *R*_s_ is the intrinsic resistance that is dominant in the high frequency range, which is the internal resistance dominated by the electrical resistivity of the electrode materials. The charge transfer resistance (*R*_ct_) at interfaces between the electrodes and electrolyte is extensively applied to electrochemical capacitor systems. *C*_dl_ is the electric double layer capacitance, which arises from the surface ion adsorption. W is the diffusion resistance when the resistance is diffusion‐dominated, which obeys the Warburg diffusion law, representing the diffusion ability of ions in electrode materials.^[^ ^45^ ^]^ Hence, fitting the high‐frequency curve yields an internal resistance (*R*_s_) of 0.6 Ω cm^2^ and an *R*_ct_ of 2.0 Ω cm^2^. Such low *R*_s_ values imply excellent intrinsic conductivities owing to the in situ grown KCo_1.3_(OH)_3.6_ on NF with K^+^ ions. Conversely, a low *R*_ct_ benefits from the powerful diffusion capacity of amorphous KCo_1.3_(OH)_3.6_.

### 2.3 Energy Storage Performance of an MAB‐KCo_1.3_(OH)_3.6_@Ni//AC Hybrid Device

**Figure 4:**
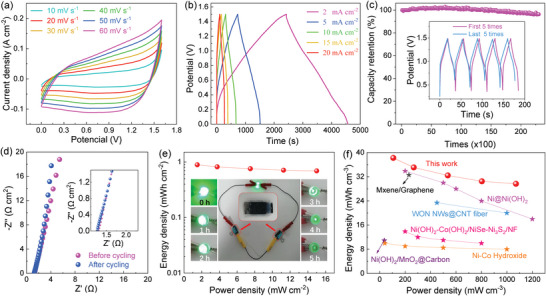
Electrochemical performances of the all‐solid‐state hybrid device (AHD) based on a MAB‐KCo_1.3_(OH)_3.6_ cathode and an activated carbon (AC) anode. a) CV curves. b) GCD curves. c) Cycling stability performances of AHD at 200 mA cm^−2^. The inset shows the first and last 5 cycles of the GCD curves. d) Nyquist plots of the AHD impedance before and after cycling. e) Areal energy density versus areal power density curves of the AHD. A lighting demonstration of the AHD as a function of the lighting duration (inset). f) Comparison of the volumetric energy and volumetric power density curves of the MAB‐KCo_1.3_(OH)_3.6_ AHD.

To further evaluate the application potential of the MAB‐KCo_1.3_(OH)_3.6_ as an energy storage device, an all‐solid‐state hybrid device (AHD) was fabricated using MAB‐KCo_1.3_(OH)_3.6_ as the cathode, activated carbon (AC)/PVDF as the anode,^[^ ^55^ ^]^ and an all‐solid PVA gel with dissolved KOH as the electrolyte, that is, constructing a KCo_1.3_(OH)_3.6_@Ni//AC AHD. The electrochemical properties are demonstrated in **Figure** **4**. CV curves of the individual KCo_1.3_(OH)_3.6_ cathode and AC are shown in Figure S9, Supporting Information with the mass balanced. Figure 4a displays the typical CV curves of an AHD that has a voltage window of up to 1.5 V, which is in good agreement with the working potential windows of the separate electrodes with respect to the oxidation and reduction potentials. The CV curves present a non‐rectangular shape with a couple of broad reversible redox peaks, suggesting a main contribution from the redox reaction. The GCD curves show a symmetrical charge/discharge process with a relatively long duration of 4600 s at a current density of 2 mA cm^−2^ (Figure 4b). According to the GCD curves, the Vol. SC of the full AHD is calculated to be 1.2 and 0.92 mAh cm^−2^ at current densities of 2 and 20 mA cm^−2^, which correspond to a Vol. SC of 51.54 and 39.44 mAh cm^−3^, respectively (Figure S10, Supporting Information). Figure 4c shows the long‐term cycling stability of the KCo_1.3_(OH)_3.6_@Ni//AC AHD after 23 000 cycles at a current density of 200 mA cm^−2^, at which a high capacity retention of 96.3% is maintained. Furthermore, the Rs (1.3 Ω cm^2^) and *R*_ct_ (20.9 Ω cm^2^) obtained from the EIS exhibit no obvious changes (Figure 4d), which also indicates the structurally robust stability for MAB‐KCo_1.3_(OH)_3.6_ during cycling. In practical terms, a float‐voltage test was conducted at 1.5 V for 72 h and it was determined that the full cell has an excellent stability with a capacity retention of 87.8% (Figure S11, Supporting Information).

The Ragone plot of the energy density versus the power density of the AHD calculated from the GCD curves is also demonstrated. Figure 4e shows an areal energy density of 0.9 mWh cm^−2^ at a power density of 1.5 mW cm^−2^, which was slightly reduced to 0.7 mWh cm^−2^ when the power density was increased to 15 mW cm^−2^. Notably, the volume of the current collector (foam Ni) is considered for a real AHD when evaluating the energy density. However, many similar studies excluded the current collector and consequently exhibited much higher energy densities.^[^ ^6^, ^14^ ^]^ Figure 4f demonstrates the VED of a whole

AHD, including the active matter KCo_1.3_(OH)_3.6_, electrolyte, AC, and foam Ni current collector. An ultrahigh VED of 38.2 mWh cm^−3^ was achieved at a power density of 107.1 mW cm^−3^, which was well maintained (30 mWh cm^−3^) at a high‐power density (1071.4 mW cm^−3^). The performance of the present MAB‐KCo_1.3_(OH)_3.6_ based on the AHD is superior to nearly all existing devices with high Vol. SCs, including Ni@Ni(OH)_2_ nanowires (33.9 mWh cm^−3^ at 200 mW cm^−3^),^[^ ^56^ ^]^ 3D Ni(OH)_2_/MnO_2_@carbon nanotubes (10.9 mWh cm^−3^ at 36.7 mW cm^−3^),^[^ ^57^ ^]^ hierarchical bimetallic hydroxide/chalcogenide core‐sheath microarrays with a Ni(OH)_2_‐Co(OH)_2_/NiSe‐Ni_2_S_3_/NF electrode (13.9 mWh cm^−3^ at 200 mW cm^−3^),^[^ ^58^ ^]^ hierarchical Ni–Co hydroxide petals with graphene petal foam (10 mWh cm^−3^ at 40 mW cm^−3^),^[^ ^45^ ^]^ flexible MXene/graphene films (32.6 mWh cm^−3^ at 230 mW cm^−3^),^[^ ^59^ ^]^ and tungsten oxynitride (23.4 mWh cm^−3^ at 450 mW cm^−3^).^[^ ^60^ ^]^ Therefore, it can be concluded that the KCo_1.3_(OH)_3.6_‐based ADH exhibits considerable superiority to previously reported devices in terms of its whole‐device VED. This is likely due to the mesoporous, amorphous, and bulk structural natures of the KCo_1.3_(OH)_3.6_ electrode material, in which the OH^−^ species could diffuse into the bulk inner without hindrance to fulfill the electrochemical redox reaction with the electroactive Co^2+^ cations and the electrons could easily transport through the whole bulk because of the structural continuity. To further evaluate the application potential of the MAB‐KCo_1.3_(OH)_3.6_ electrode, two AHD components were connected in series to power a green light (VF = 3.0 V, P = 60 mW), which lasted for over 5 h after being charged for 5 s, as shown in Figure 4e‐inset, demonstrating its considerable application potential.

## 3 Discussion

High electron conduction and fast ion diffusion are the two most crucial characteristics for achieving high‐performance electrochemical capacitors. In fact, these two characteristics are often contradictory mutually. For example, exquisite nanostructures afford sufficient ionic transportation for electrochemical redox reactions but hinder electron conduction due to the abundance of surfaces and interfaces, while the large‐size (bulk) materials maintain the structural continuity for electron transportation but constrain the ionic diffusion. To breakthrough these categories, a simultaneous mesoporous, amorphous, bulk (MAB) strategy is much promising. A “mesoporous” route, which has been extensively adopted previously, is maintained to mainly improve internal surface/interfacial utilization with the capable high specific areas; while, “amorphous” is to improve the interior utilization and ionic conductivity. When the electrode materials fall into a relatively large‐scale size (for instance the bulk materials), these well‐designed materials (mesoporous and amorphous structures) would not exert the effects, because the available ionic diffusion and active sites are drastically reduced. This is why a bulk material electrode hardly achieves high active performance. The benefit from a bulk material is the high structural‐continuity for electron delivering as well as the high structural‐stability and robustness during the electrochemical redox compared to any nanoscale materials or exquisite nanostructures. It is, thus, apparently rather challenging to achieve high activity in a bulk material.

**Figure 5:**
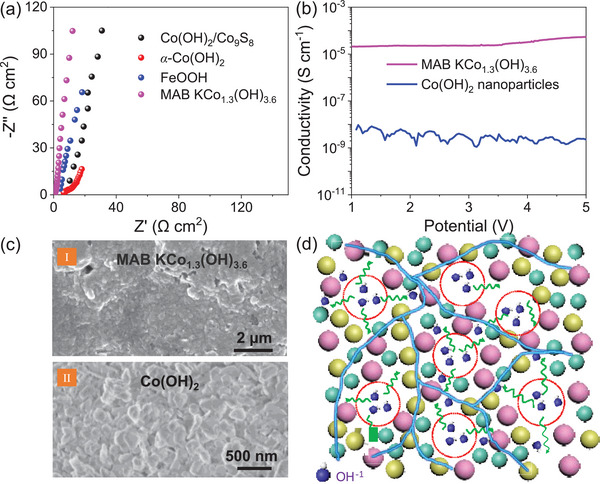
Ion and electron transportation in MAB‐KCo_1.3_(OH)_3.6_. a) Complex impedance comparison of MAB‐KCo_1.3_(OH)_3.6_ and crystalline hydroxide. b) Conductivity comparison of MAB‐KCo_1.3_(OH)_3.6_ and Co(OH)_2_ nanoparticles. c) SEM images of MAB‐KCo_1.3_(OH)_3.6_ and Co(OH)_2_. d) Ionic diffusion and electron transport during electrochemical a redox reaction. The balls denote the atoms and the solid‐blue lines denote the electron transportation paths.

To gain insights into the ionic/electron transportation, **Figure** **5**a compares the complex impedance plots of MAB‐KCo_1.3_(OH)_3.6_ with crystalline congener hydroxides, including Co(OH)_2_/Co_9_S_8_,^[^ ^37^ ^]^ *α*‐Co(OH)_2_,^[^ ^41^ ^]^ and FeOOH.^[^ ^61^ ^]^ MAB‐KCo_1.3_(OH)_3.6_ possesses a lower diffusion resistance and has a calculated ion conductivity as high as 4.6 × 10^−2^ S cm^−1^. This reflects the significant superiority of the MAB structural features for ionic delivery as the proton conductivity in a material is generally lower than 10^−3^–10^−5^ S cm^−1^ in an ambient environment.^[^ ^62^ ^]^ Moreover, MAB‐KCo_1.3_(OH)_3.6_ also exhibits a high electron conductivity (>10^−5^ S cm^−1^) under a high mass loading (11.7 mg cm^−2^) that is 3–4 orders higher than that of typical high‐performance Co(OH)_2_ nanoparticles (Figure 5b). This is likely attributed to the bulk feature (Figure 5c‐inset I) of MAB‐KCo_1.3_(OH)_3.6_. In addition, the intrinsic conductivity is improved by introducing K^+^ species.^[^ ^1^ ^]^ However, the grain boundaries in Co(OH)_2_ (Figure 5c‐inset II) will lead to the formation of a large barrier layer that would reduce the electron mobility.^[^ ^63^ ^]^ Consequently, the bulk nature significantly enhances the conductivity of the whole electrode by eliminating the abundant interfaces, regardless of whether the intrinsic conductivity is improved. This is also the reason why most nanomaterials cannot achieve satisfactory electrochemical performances at high mass loadings.

To emphasize the superiority of the MAB strategy, Figure 5d demonstrates the electron/ionic transportation of the unimpeded delivering pathways. The mesoporous structure allows the electrode materials to be fully immersed in and/or easily touch the electrolyte, which in this study was 6 m KOH (highlighted by dash cycles). Moreover, the cations and anions in the amorphous solids are arranged in disordering and have a high probability of creating atom deficiencies, which would facilitate the OH^−^ ion diffusion into the inner bulk for the electrochemical redox reaction (Figure 2c and Figure S4d, Supporting Information‐inset). The structural (chemical bonds) continuity of the bulk materials could benefit from delivering electrons (highlighted by solid lines) without being hindered by the surfaces or interfaces; this would also improve the stability of the bulk material (Figure 2a). Therefore, the proposed MAB route (Figure 1) not only resolves the aforementioned two contradictions but also ensures the structural stability of the device during electrochemical redox reactions and high‐efficient utilization of the active sites.

## 4 Conclusion

This study used a MAB strategy to improve ion/electron transport, decrease the dead‐volume, ensure cycling stability, and retain the sound electrochemical performance of electrodes at high mass loadings. An in situ‐grown MAB‐KCo_1.3_(OH)_3.6_ was fabricated on the Ni slice (KCo_1.3_(OH)_3.6_@Ni) of an electrochemical capacitor and showed a high Areal SC (1.74 mAh cm^−2^), high Mass SC (148 mAh g^−1^) under a high mass loading (11.7 mg cm^−2^), excellent cycling stability, and high Vol. SC (123.7 mAh cm^−3^). The fabricated AHD with KCo_1.3_(OH)_3.6_@Ni and AC achieved an excellent VED of 38.2 mWh cm^−3^ at a power density of 107.1 mW cm^−3^, which is superior to that of existing high‐performance materials and devices. Therefore, the MAB strategy could be adapted for the design of a broad range of electrode materials under high mass loadings to significantly promote the practical applications in the electrochemical capacitor field.

## Conflict of Interest

The authors declare no conflict of interest.

## Supporting information

Supporting Information

## Data Availability

The data that support the findings of this study are available from the corresponding author upon reasonable request.
