# Supplementary material for: Mesoporous Amorphous Bulk Material Electrodes for Ultrahigh Volumetric Capacity Electrochemical Capacitors
Source: Adv Sci (Weinh). 2023 May 3;10(20):2300727. doi: 10.1002/advs.202300727 (PMC10369296; doi:10.1002/advs.202300727)
Supplement: Supplementary file 1 — Supporting Information [file ADVS-10-2300727-s001.pdf]

ADVANCED SCIENCE
Open Access

Supporting Information

for *Adv. Sci.*, DOI 10.1002/advs.202300727

Mesoporous Amorphous Bulk Material Electrodes for Ultrahigh Volumetric Capacity Electrochemical Capacitors

*Lun Li, Changjin Guo, Shuangbao Wang and Wanbiao Hu**

Supplementary Information

Mesoporous amorphous bulk material electrode for ultrahigh volumetric capacity electrochemical capacitors

Lun Li,[a] Changjin Guo,[a] Shuangbao Wang,[a,b] and Wanbiao Hu*[a,b]

[a] L. Li, C. Guo, Dr S. Wang, Prof W. Hu

Key Laboratory of LCR Materials and Devices of Yunnan Province
National Center for International Research on Photoelectric and Energy Materials
School of Materials and Energy
Yunnan University
Kunming 650091, P. R. China
E-mail: huwanbiao@ynu.edu.cn

[b] Dr S. Wang, Prof W. Hu

Electron Microscopy Center

Yunnan University

Kunming 650091, P. R. China

Experimental section

Synthesis of $KCo_{1.3}(OH)_{3.6}$@Ni foam (NF). The mesoporous amorphous bulk (MAB) electrode material $KCo_{1.3}(OH)_{3.6}$ was electrodeposited through a standard three-electrode system by using a Hg/HgO reference electrode and electrochemical workstation. The MAB were deposited on Ni foams (3×3 cm) (pretreated using a HCl solution to remove Ni oxides from the surface) at -1 V for 5000 s in an electrolyte containing 120 mM Co^{2+} and K^{+} nitrates (Co : K=1 : 1). On average, the mass loading for the $KCo_{1.3}(OH)_{3.6}$ is 11.7 mg cm^{-2}, and the mole ratio of Co^{2+}: K^{+}=1.3: 1 (Table S1) that is determined by the ICP-AES measurement. It is noted that the calculation of the loaded mass of the active matters $KCo_{1.3}(OH)_{3.6}$ is based on the ICP-AES result (the calculation details are seen in Supplementary Figure S12). The deposited $KCo_{1.3}(OH)_{3.6}$@Ni was directly used as cathode for electrochemical capacitor fabrication.

Fabrication of AC electrode. The activated arbon (AC) electrode was prepared by mixing the active carbon hybrid with carbon black and polyvinidenefifuoride (PVDF) as the weight ratio of 8: 1: 1. Then, N-methy1-2pyrrolidone (NMP) was dropped into the mixture and thoroughly stirred for 30 min to form the coating slurry. This slurry was pasted onto a piece of nickel foam and the dried at 80 ^{o}C in an oven for 24 h. The dried nickel foam was pressed to form a thin foil at a pressure of 10 Mpa for 30 s.

Fabrication of $KCo_{1.3}(OH)_{3.6}$@Ni//AC all-solid-state hybrid device (AHD).

The hybrid device was assembled by using the $KCo_{1.3}(OH)_{3.6}$@Ni electrode as the positive electrode and activated carbon (AC) on Ni foam as the negative electrode. To meet the requirements of practical applications, the all-solid-state hybrid device was fabricated with the solid electrolyte. Specifically, 2.5 g Poly (vinyl alcohol) (PVA) was soaked in 50 ml distilled water for 2 h, and heated in a 50 ^{o}C water bath for 1 h, and

then the water bath temperature was raised to 90 °C. After PVA was completely dissolved, 20 ml of 5 M KOH solution was added and stirred for 30 min. The solid electrode consists of three layers, the upper and lower layers are $KCo_{1.3}(OH)_{3.6}$@Ni battery-like positive electrode and AC negative, respectively, while the middle-layer filter paper diaphragm is used to separate the positive and negative electrodes. The mass ratio of negative electrode (AC) to the positive electrode ($KCo_{1.3}(OH)_{3.6}$) was decided based on the charge balance theory ($q^-=q^+$). To get $q^- = q^+$, the mass balancing is calculated according to formula:

$$\frac{m_+}{m_-} = \frac{C_- \times \Delta V_-}{C_- \times \Delta V_+}$$

And the actual mass loading of AC is 15.1 mg cm^{-2}.

Characterization analysis techniques. All the characterizations were adopted mesoporous amorphous bulk $KCo_{1.3}(OH)_{3.6}$ pressed in 10 Mpa after 30 seconds.

The microstructure and morphology were examined by field emission scanning electron microscopy (FESEM, Gemini 500) and transmission electron microscopy (TEM, Talos 200X). The ratio of the elements of the sample was detected by inductively coupled plasma atomic emission spectrometer (ICP-AES, PlasmaQuant PQ9000). The chemical valence states of the elements were analyzed by X-ray photoelectron spectroscopy (XPS, K-Alpha+). The BET specific surface area and pore size were characterized by accelerated surface area and porosimetry system (BET, ASAP 2460). The conductivities were examined by a high resistance meter (Keithley 6517B). Atomic configurations for Fourier transform k^3x (k) were analyzed by X-ray absorption fine structure (XAFS) spectroscopy (RapidXAFS, 1 M).

Electrochemical measurements. The electrochemical properties *e.g.* cyclic voltammetry (CV), galvanostatic charge-discharge (GGD) and electrochemical impedance (EIS) *etc.* were evaluated by an Electrochemical workstation (CHI660E,

CH instrument, Shanghai Chenhua). A classic three-electrode method was adopted to measure the above CV, GGD and EIS, where the afore-fabricated MAB-$KCo_{1.3}(OH)_{3.6}$@Ni was used as working electrode, a platinum as the counter electrode, a calomel electrode as the reference electrode, and a 6 mol/L KOH solution as the electrolyte. Before measurement, the working electrode was firstly immersed into the electrolyte for 30 minutes to make completely wetted, which then was performed the CV test for 20 rounds to fully activate the electrode material.

The areal capacity of MAB-$KCo_{1.3}(OH)_{3.6}$@Ni samples was calculated from the GCD curves using the following equation:

$$Q_{ac} = \frac{i\int \Delta V d\Delta t}{3.6 \times a\Delta V} \qquad (1)$$

Where Q_{ac} is the areal capacity (mAh cm^{-2}), *i* is the discharge current (A), Δt is the discharge time(s) ΔV is the potential window (V), and *a* is the area of the electrode (cm^2). The volumetric capacities (Vol. SC) (Q_v, mAh cm^{-3}) were obtained from the areal capacity with following equation: $Q_v = Q_{ac} \times 10000\ \mu\text{m}/140\ \mu\text{m}$ (note: the thickness of an individual MAB-$KCo_{1.3}(OH)_{3.6}$@Ni electrode is about 140 μm (0.14 mm) that can be seen in Figure 3d), where $10000\ \mu\text{m}/140\ \mu\text{m}$ is the number of electrode slices per cubic centigrade. For all-solid-state hybrid device (AHD), the capacities were calculated using the following formula: $Q_v = Q_{ac} \times 10000\ \mu\text{m}/(140\ \mu\text{m} + 211\ \mu\text{m})$, where 211 μm is the thickness of the AC negative electrode. It is noted that all the electrode slices were pressed under 10 Mpa for 30 seconds.

The areal energy and power densities of the MAB-$KCo_{1.3}(OH)_{3.6}$@Ni//AC device was estimated using the following equations:

$$E_d = \frac{1}{7.2} Q_{ac} \Delta V \qquad (2)$$

$$P_d = \frac{E_d}{\Delta t} \times 3600 \qquad (3)$$

Where Q_{ac} is the areal capacity (mAh cm^{-2}) calculated from EQ(1), E_d is the areal energy density (mWh cm^{-2}), ΔV is the potential window (V), P_d is the areal power density (W cm^{-2}), and Δt is the discharge time (s). The volumetric energy density and power density were obtained relying on areal capacity.

The ion conductivity (σ) is calculated by the formula $\sigma = \frac{L}{RS}$ based on the Nyquist plots of the complex impedance, where *L* is thickness of the electrode, *R* is the resistance obtained from the equavelent-circuit fitting, and *S* is contacting area.

The electronic conductivities (σ) of the pressed slices for MAB-$KCo_{1.3}(OH)_{3.6}$ and $Co(OH)_2$ nanoparticle powders are calculated by the formula $\sigma = \frac{IL}{US}$, where *L* is the thickness of electrode (cm), *I* is the is the current (A), *U* is the potencial (V) and *S* is the area of electrode.

Table S1. The ratio of Co and K of the sample tested by ICP.

Element	Constant volume/ml	Dilution factor	Instrument reading (mg/L)	Relative molarity (mol)
Co	50	50	2.4416	1.331
K	50	50	1.2171	1

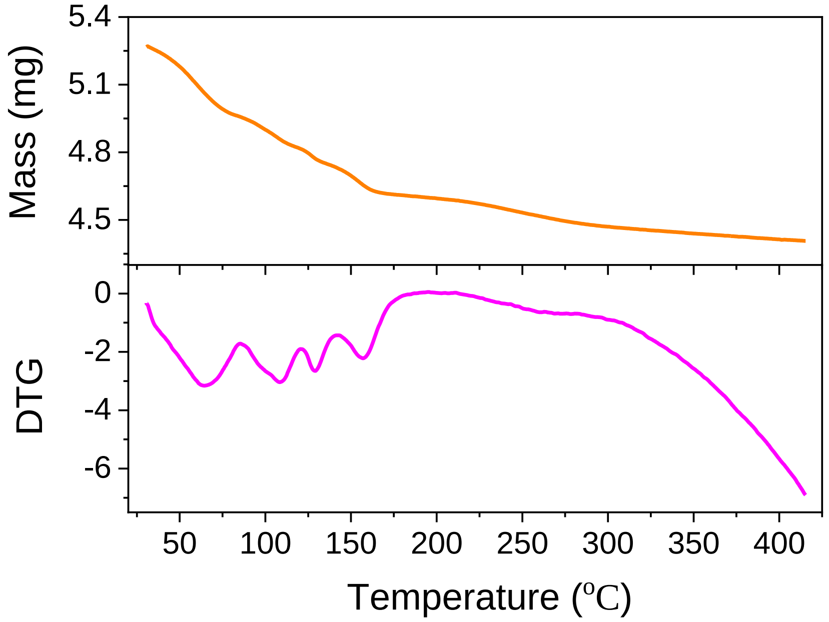

Figure S1. Thermogravimetric (TG) the differentiated TG (DTG) curve of $KCo_{1.3}(OH)_{3.6}$. The mass loss is in a homogeneous process, while crystalline $Co(OH)_2$ presents a trong weight loss in 295 °C (Journal of Colloid and Inergace Scienc, DOI: 10.1016/j.jcis.2013.01.070), which is far different from the weight loss curve of $KCo_{1.3}(OH)_{3.6}$, suggesting that the atomic arrangement may be disorder for $KCo_{1.3}(OH)_{3.6}$.

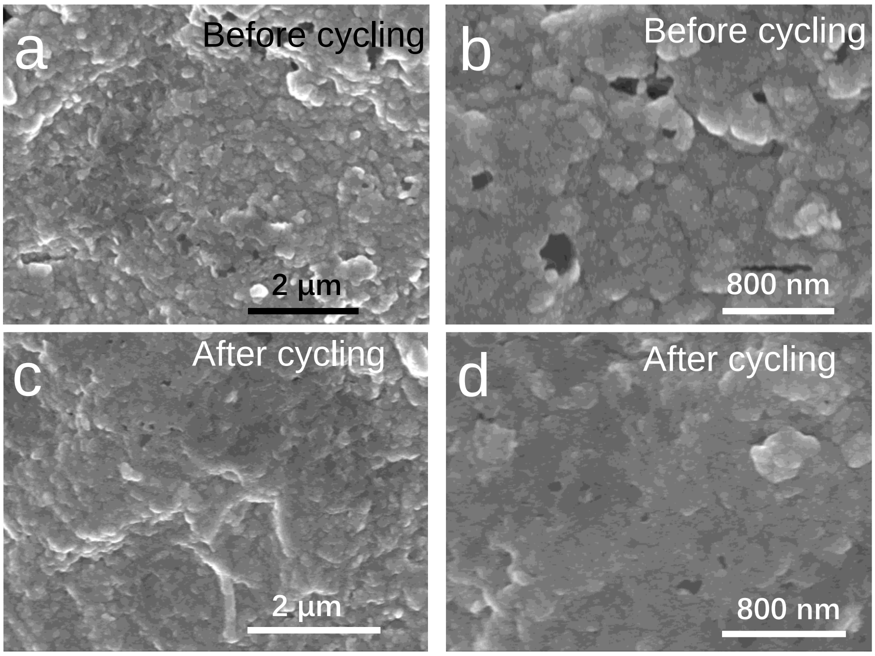

Figure S2. Morphology characterizations of mesoporous amorphous bulk (MAB) $KCo_{1.3}(OH)_{3.6}$ before and after cycling. (a-b) SEM image of MAB-$KCo_{1.3}(OH)_{3.6}$ before cycling. (b-d) SEM images of MAB-$KCo_{1.3}(OH)_{3.6}$ after 4000 times cycling. The particle size is quite large, far beyond 10 μm, and the morphology was almost unchanged despite the long cycle, confirming that the bulk feature can guarantee the structural and chemical stability .

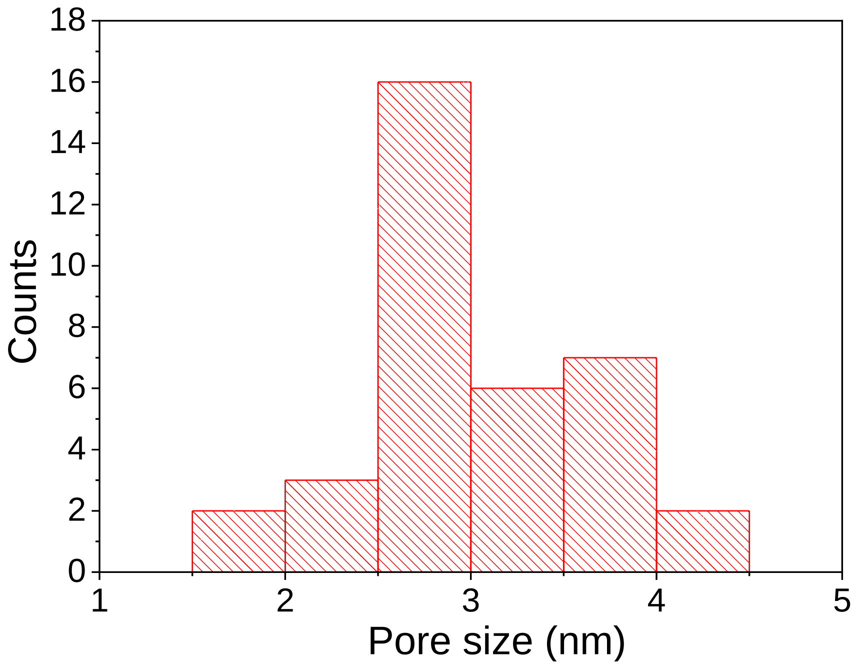

Figure S3. Pore size distribution analysis of MAB-K$Co_{1.3}(OH)_{3.6}$. The pore sized distribution was counted by photoshop software based on TEM image (Figure 2b), which corresponds well with the pore size distribution characterized with Barrett-Joyner-Halenda (BJH) analysis method (Figure 2d), confirming that the pores are uniformly distributed at 2~4 nm, which enable the materials can be fully immersed, increasing the surface utilization and decreasing the ions transport distance.

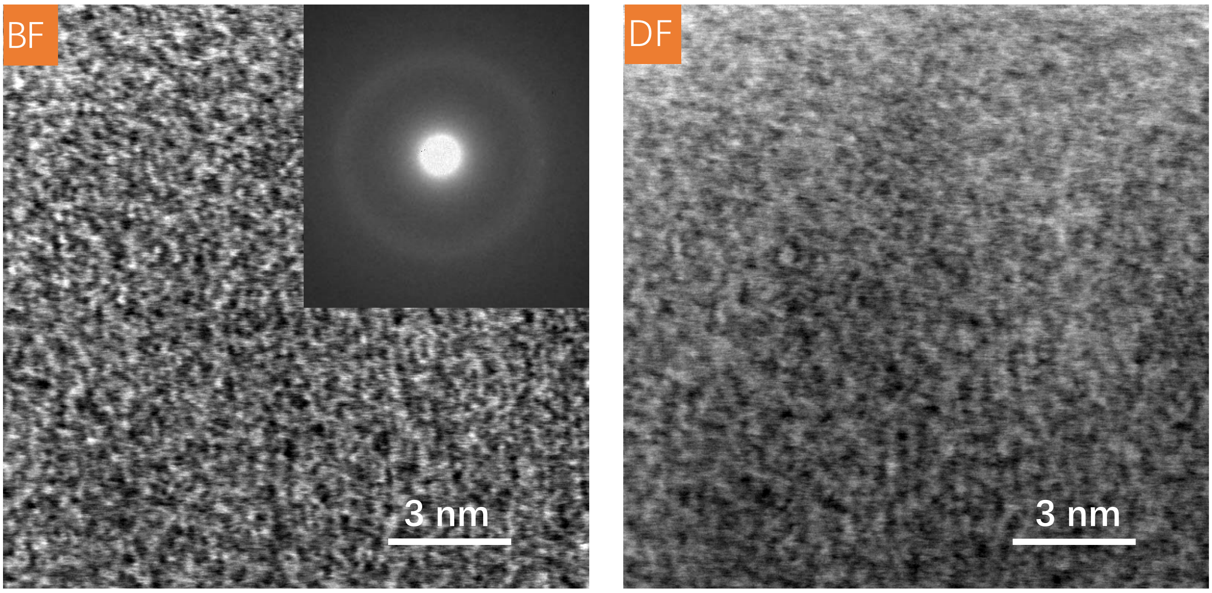

Figure S4. HRTEM images from the BF and DF observations. HRTEM-BF verifies the amorphous natures characterized by the disordered atomic arrangement and vague SAED ring. Therefore, there may exist a huge set of atomic deficiency *e.g.* perhaps cation defects and/or vacancies, which can facilitate the OH^- diffusion in the inner materials and redox.

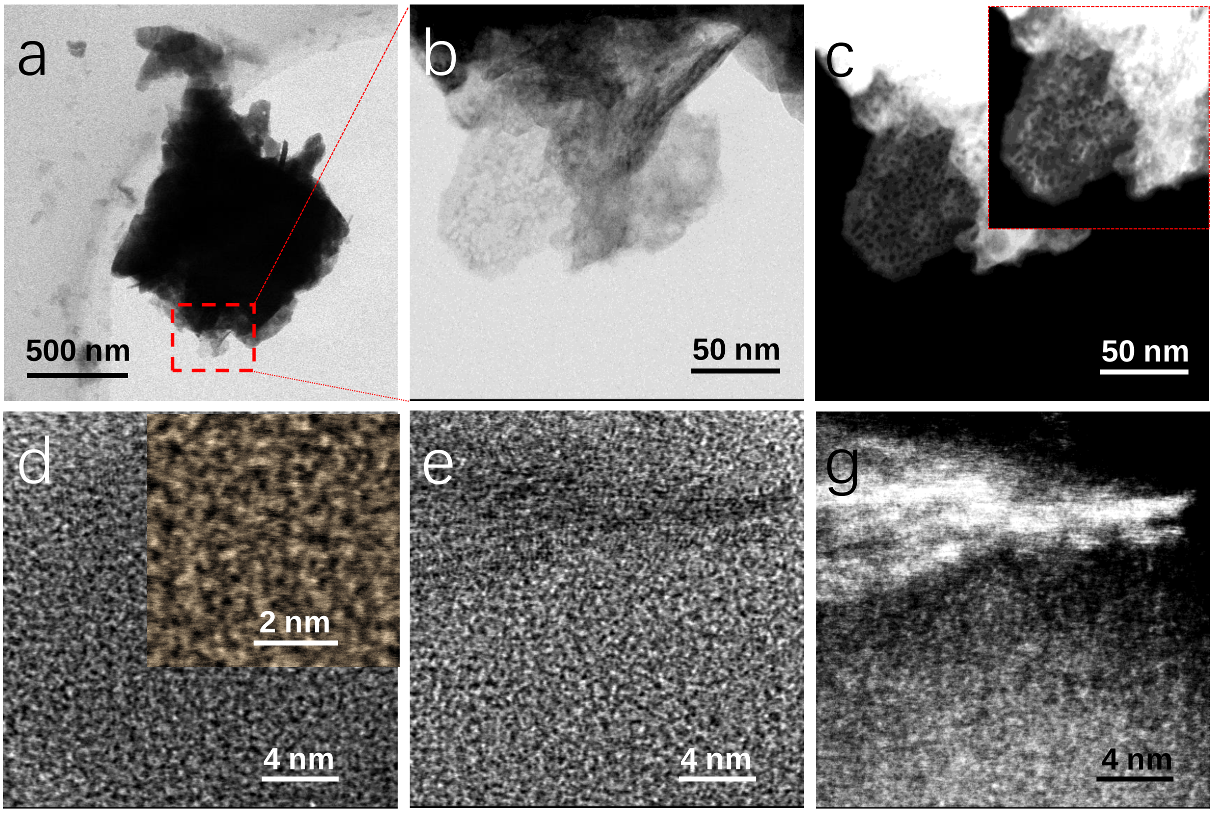

Figure S5 Structure and morphology characterizations. (a) TEM-BF image. (b) HRTEM-BF image. (c) STEM-HAADF image. There exist a huge amount of the pores. (d) Atomic-resolution TEM-BF image. It indicates that the atomic arrangement is fully in disordered. (e-g) Comparasion of HRTEM-BF/DF images. The overall particle size is rather large. Thus, one sample with the size still more that 500 nm (Figure 5a) is chosen for the TEM characterization. From these TEM charaterizations of different scales, the disordered atomic arrangement and deficiencies are shown clearly as regularly periodic atom distributions can not be observed, which well mathches the amorphous feature.

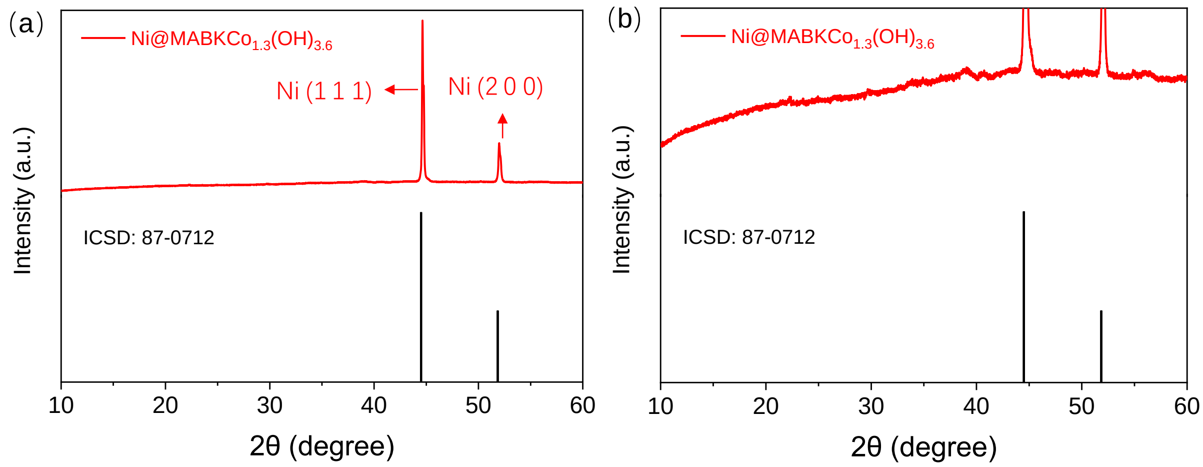

Figure S6. XRD pattern of MAB-$KCo_{1.3}(OH)_{3.6}$@Ni. (a) Original XRD pattern of MAB-$KCo_{1.3}(OH)_{3.6}$@Ni. (b) Magnified XRD pattern of MAB-$KCo_{1.3}(OH)_{3.6}$@Ni. The original XRD does not show any signals of MAB-$KCo_{1.3}(OH)_{3.6}$, only two diffraction lines of the Ni denoting (1 1 1) and (2 0 0) respectivily. Furthermore, magnified XRD pattern of MAB-$KCo_{1.3}(OH)_{3.6}$@Ni only shows some fluctuation without any clear peaks. These results indicate that MAB-$KCo_{1.3}(OH)_{3.6}$ blongs to an amorphous material.

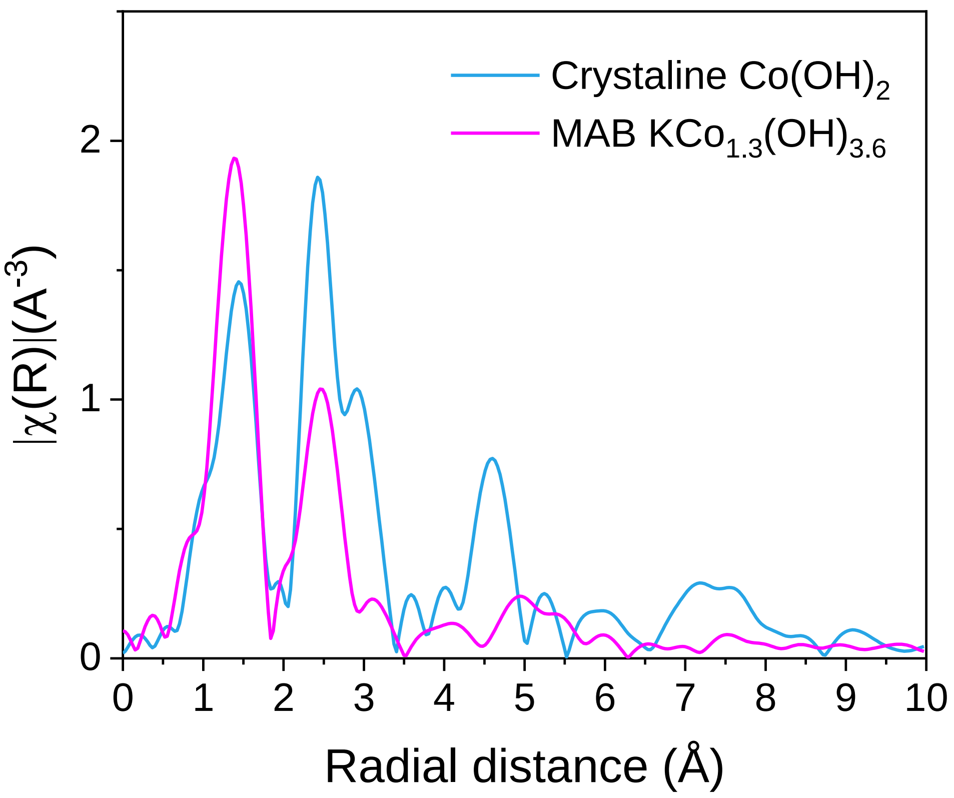

Figure S7. XAFS spectra for Fourier transforn $k^3x(k)$ of Co K-edge. The sepctrum of MAB-$KCo_{1.3}(OH)_{3.6}$ demonstrates a short-range order while long-range disorder atomic configuration, but the compared sample *i.e.* the crystalline $Co(OH)_2$ shows an obvious long-range atomic ordering. This suggests from the atomic scale again that the MAB-$KCo_{1.3}(OH)_{3.6}$ should belong to an amorphous material.

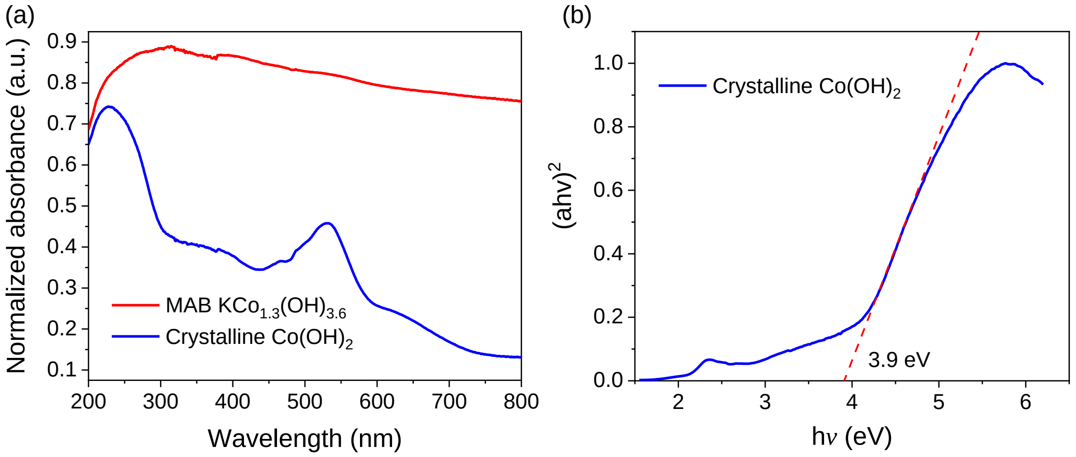

Figure S8. UV-vis diffuse-reflection absorption spectra. (a) UV-vis diffuse-reflection absorption spectra of MAB-$KCo_{1.3}(OH)_{3.6}$ and crystalline $Co(OH)_2$. (b) Plot of $(ahv)^2$ *vs.* (hv) based on the direct optical band gap for crystalline $Co(OH)_2$. The MAB-$KCo_{1.3}(OH)_{3.6}$ presents a full adsorption in visible-light region from the UV-vis spectrum, indicating an excellent electronic conduction. While, crystalline $Co(OH)_2$ shows a relative low adsorption in visible-light region due to the d-d electron transition, but it has a relatively large band gap of about 3.9 eV (Figure S8b), indicating that normal crystalline $Co(OH)_2$ is limited in the excellent electronic transport during redox process.

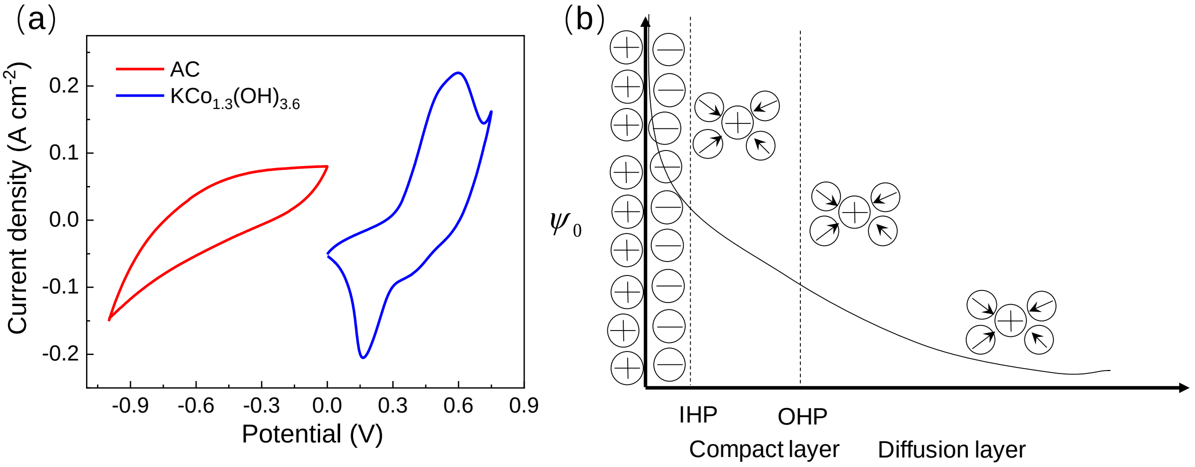

Figure S9. (a) CV curves of the activated carbon (AC) and MAB-$KCo_{1.3}(OH)_{3.6}$ at a scan rate of 25 mV s^{-1}, respectively. (b) Diagram of Grahame's electric double layer theory. The CV curve of MAB-$KCo_{1.3}(OH)_{3.6}$ shows a clear redox peak, which should belong to a battery-type material. The CV curve of AC does not exhibit a standard rectangular shape, which is due to the high mass loading (15 mg cm^{-2}) of AC. Based on Grahame's electric double layer theory (Figure 9b), it is extramely hard (or even impossible) for a specific real material to afford a standard rectangle CV shape, because it is strongly affected by the diffusion layer and compact layer, which generally results in a nonstandard rectangle shape. Furthermore, the phenomenon will be more obvious with increasing the AC thickness. The mass loading of AC is about 15 mg cm^{-2}, leading to the nonstandard rectangle.

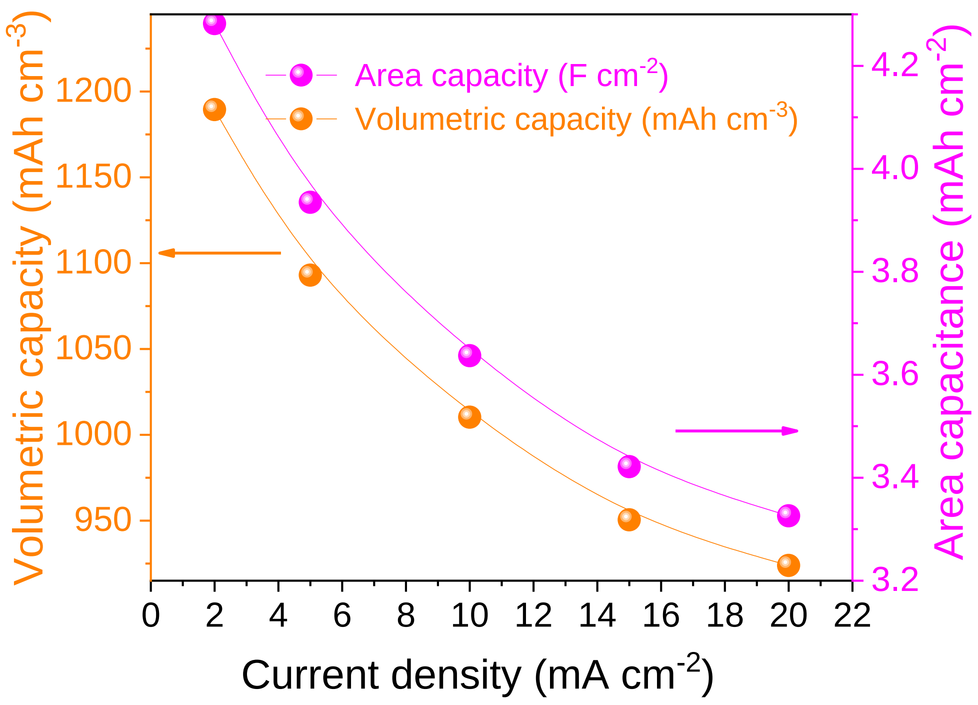

Figure S10. The full ASC device delivers area capacity of 1.2 mAh cm^{-2} and 0.92 mAh cm^{-2} at current density of 2 and 20 mA cm^{-2}, respectively, corresponding to a volumetric capacity (51.54 mAh cm^{-3}) and 39.45 mAh cm^{-3}. The ASC device displays a good rate capability with 77.58% of capacity retained in the current density range from 2 to 20 mA cm^{-2}.

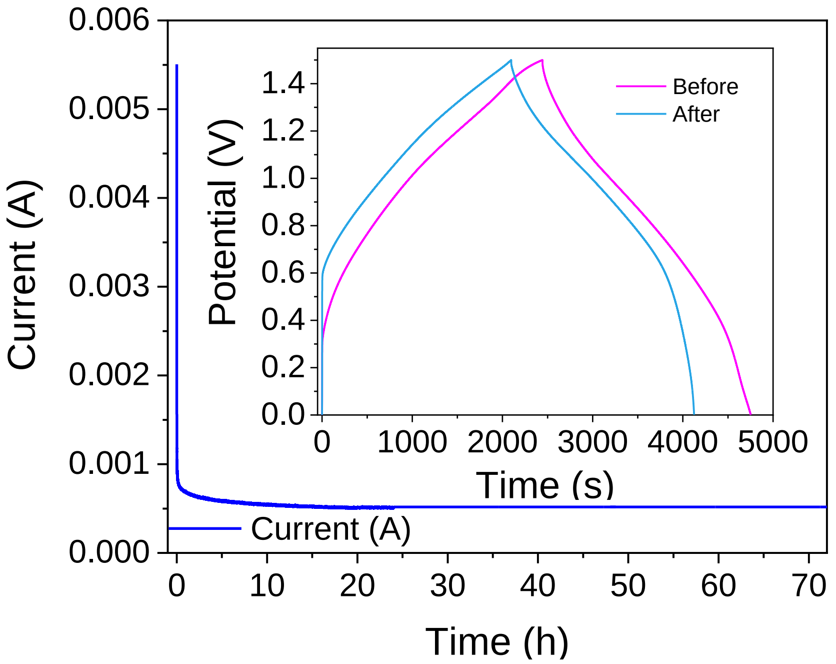

Figure S11. Current of float-voltage testing for asymmetric cell under 1.5 V. The inset shows the GCD curves before and after float-voltage testing, which perform an excellent stability with capacity retention 87.8% after 72 hours constantly floating-voltage testing under 1.5 V. The excellent stability should attribute to the bulk feature of MAB-$KCo_{1.3}(OH)_{3.6}$. Although the float-voltage testing under 1.5 V is doomed to erode electrode materials, bulk feature enables electrode materials more stable.

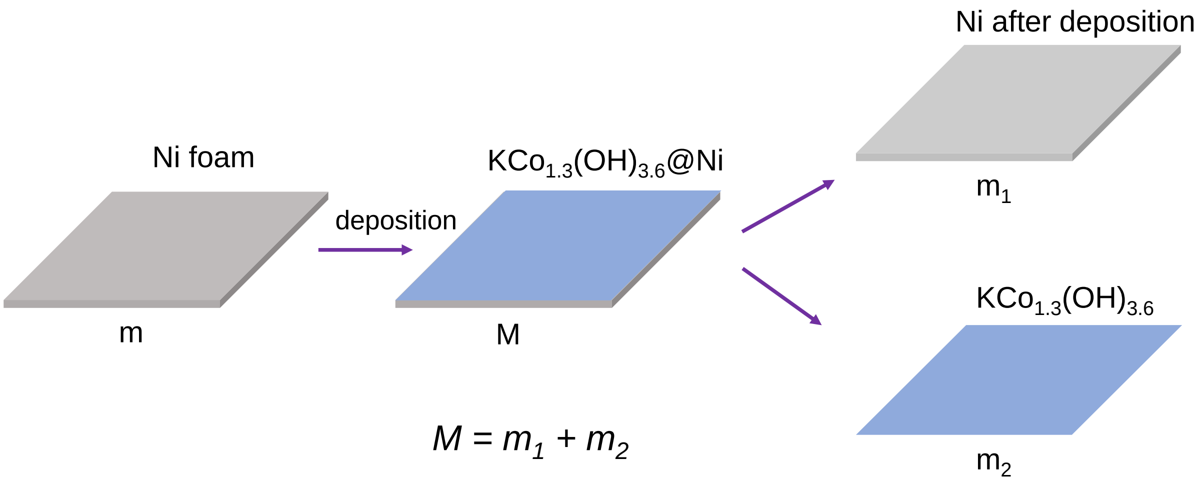

Figure S12. Mass loading calculation of MAB-$KCo_{1.3}(OH)_{3.6}$. A whole MAB-$KCo_{1.3}(OH)_{3.6}$@Ni was dissolved fully for ICP-AES measurement (supplementary Table 1). Generally, the mass for the Ni before and after deposition (reaction) may not be same, i.e. $m_1 \neq m$; otherwise, the mass of the active matter ($KCo_{1.3}(OH)_{3.6}$) should be easily obtained by $m_2 = M - m$, where M (the mass of the whole MAB-$KCo_{1.3}(OH)_{3.6}$@Ni) can be directly weighted. Anyway, to the get the accurate mass loading of the MAB-$KCo_{1.3}(OH)_{3.6}$, the calculation can be performed by the following equation,

$$m_2 = \frac{1}{\frac{1.3M_{Ni}}{kM_{MAB}} + 1} M$$

where M_{Ni} (=58.7) is the molar mass of Ni, M_{MAB} (=176.9) is the molar mass of $KCo_{1.3}(OH)_{3.6}$, M is the mass of the whole MAB-$KCo_{1.3}(OH)_{3.6}$@Ni) which can be directly weighted by a balance, and *k* is the atomic ratio of Co : Ni, which can be determined by ICP-AES measurement.
